# Antimicrobial GL13K Peptide Coatings Killed and Ruptured the Wall of Streptococcus gordonii and Prevented Formation and Growth of Biofilms

**DOI:** 10.1371/journal.pone.0111579

**Published:** 2014-11-05

**Authors:** Xi Chen, Helmut Hirt, Yuping Li, Sven-Ulrik Gorr, Conrado Aparicio

**Affiliations:** 1 Minnesota Dental Research Center for Biomaterials and Biomechanics, Department of Restorative Sciences, University of Minnesota School of Dentistry, Minneapolis, Minnesota, United States of America; 2 Department of Diagnostic and Biological Sciences, University of Minnesota School of Dentistry, Minneapolis, Minnesota, United States of America; University of Oklahoma Health Sciences Center, United States of America

## Abstract

Infection is one of the most prevalent causes for dental implant failure. We have developed a novel antimicrobial peptide coating on titanium by immobilizing the antimicrobial peptide GL13K. GL13K was developed from the human salivary protein BPIFA2. The peptide exhibited MIC of 8 µg/ml against planktonic Pseudonomas aeruginosa and their biofilms were reduced by three orders of magnitude with 100 µg/ml GL13K. This peptide concentration also killed 100% of Streptococcus gordonii. At 1 mg/ml, GL13K caused less than 10% lysis of human red blood cells, suggesting low toxicity to mammalian cells. Our GL13K coating has also previously showed bactericidal effect and inhibition of biofilm growth against peri-implantitis related pathogens, such as Porphyromonas gingivalis. The GL13K coating was cytocompatible with human fibroblasts and osteoblasts. However, the bioactivity of antimicrobial coatings has been commonly tested under (quasi)static culture conditions that are far from simulating conditions for biofilm formation and growth in the oral cavity. Oral salivary flow over a coating is persistent, applies continuous shear forces, and supplies sustained nutrition to bacteria. This accelerates bacteria metabolism and biofilm growth. In this work, the antimicrobial effect of the coating was tested against Streptococcus gordonii, a primary colonizer that provides attachment for the biofilm accretion by P. gingivalis, using a drip-flow biofilm bioreactor with media flow rates simulating salivary flow. The GL13K peptide coatings killed bacteria and prevented formation and growth of S. gordonii biofilms in the drip-flow bioreactor and under regular mild-agitation conditions. Surprisingly the interaction of the bacteria with the GL13K peptide coatings ruptured the cell wall at their septum or polar areas leaving empty shell-like structures or exposed protoplasts. The cell wall rupture was not detected under regular culture conditions, suggesting that cell wall rupture induced by GL13K peptides also requires media flow and possible attendant biological sequelae of the conditions in the bioreactor.

## Introduction

Dental implants have rapidly become the treatment of choice for patients who are in need to replace missing teeth. According to data from the American Academy of Implant Dentistry, the annual dental implant market reaches $1.3 billion in the US, $8.1 billion globally, and the numbers are still growing. Despite significant progress in clinical success rates in recent years, an 8% implant failure rate translates into more than one million failed implants per year worldwide [1] with infection being one of the most prevalent causes for implant failure. Indeed, 20% of implants with an average function time of 5 to 11 years develop peri-implantitis [2], which demonstrates the severity of the problem.

Functionalization of titanium surfaces with coatings made of antimicrobial agents has recently been explored to inhibit peri-implant infections [3]. The coatings can contain nanoparticles of pure elements [4], [5]; sanitizing agents and disinfectants [6]; and antibiotics as well as antimicrobial peptides. Gentamicin [7]–[9] and Vancomycin [10]–[13] have been coated on Ti surfaces for protecting from infection dental and orthopedic implants. Although antibiotic coatings on titanium proved to be effective in vitro and in vivo, their use is controversial because of their potential host cytotoxicity and bacterial resistance [3]. The use of antimicrobial peptides (AMPs) as an antimicrobial approach to improve implant performance has recently been introduced due to their broad-spectrum activity against bacteria, fungi and virus, low host cytotoxicity, and low bacterial resistance [14]. Different cationic antimicrobial peptides derived from human proteins have been either physically adsorbed [15], [16] or covalently attached [17] on implant surfaces. These implants displayed antimicrobial activity against pathogens related with orthopedic peri-implantitis. We have focused on developing an antimicrobial peptide coating with activity against pathogens associated with dental peri-implantitis. In our previous work, we bonded the antimicrobial peptide GL13K to titanium surfaces using silane coupling agents to produce coatings that have covalent attachment to the metallic substrate and that have significant antimicrobial activity against the Gram negative bacterium *Porphyromonas gingivalis* [18], an oral pathogen that is closely associated with the development of biofilms and dental peri-implantitis [19]. The peptide, GL13K, which was derived from the human salivary protein Parotid Secretory Protein (BPIFA2), exhibited an MIC of 8 µg/ml against planktonic Pseudonomas aeruginosa and their biofilms were reduced by three orders of magnitude with 100 µg/ml GL13K. This peptide concentration also killed 100% of Streptococcus gordonii. At 1 mg/ml, GL13K caused less than 10% lysis of human red blood cells, suggesting low toxicity to mammalian cells [20], [21]. The GL13K peptide coating showed bactericidal effect and inhibition of biofilm growth against peri-implantitis related pathogens, such as Porphyromonas gingivalis. Additionally, the coating had resistance to hydrolytic and mechanical challenges with no significant release of peptides from the titanium surface and was cytocompatible with osteoblasts and human gingival fibroblasts [18].

Most of the previous in vitro work to assess the effects of the antimicrobial coatings on the targeted bacteria has been performed under static or mild agitation culture conditions. Those culture conditions do not simulate the conditions of biofilm formation and growth in the oral cavity. In the mouth the salivary flow over the coating is persistent, applies significant shear forces, and supplies sustained nutrition to the bacteria. This may accelerate bacterial metabolism, growth and biofilm formation. Others showed that the formation and growth of Escherichia coli biofilm on surfaces in a drip flow bioreactor was twice that obtained in a shaker [22], assessed by both CFU amounts and carbohydrate and protein concentrations. This indicates that biofilm grown in the drip flow bioreactor system will be more similar to the in vivo conditions and present a greater challenge to the antimicrobial peptide coatings than standard culture conditions.

In this report, coatings with the antimicrobial peptide GL13K were tested in a drip-flow reactor against Streptococcus gordonii, a primary colonizer on oral surfaces that provides attachment for the subsequent pathogenic biofilm formation by P. gingivalis [23]. Adherence of P. gingivalis to surfaces in the developed biofilm depends on deposition of S. gordonii cells on the salivary pellicle at the colonized surface [23]. If S. gordonii is not present in the biofilm, only a few P. gingivalis cells are able to attach on the surface and as a result they are easier to detach and remove from the compromised surface [19]. Notably, S. gordonii has been found in the microbiota of bacterial colonization immediately after installation of oral implants [24] as well as on locations associated with dental peri-implantitis [25]. Therefore, strategies that prevent S. gordonii adhesion on the surface compromise the biofilm formation, and therefore can minimize the risk of developing peri-implantitis. The GL13K peptide coatings prevented S. gordonii biofilm formation on titanium disks and exerted unique effects on the mechanical integrity of the bacteria cell wall.

## Material and Methods

### Coating of Peptides

The peptide coatings on Ti surfaces were prepared as previously described [26]. Briefly, commercially pure Titanium Grade II discs were polished with a suspension of alumina particles and immersed in 5 M NaOH overnight at 60°C to activate the surface by forming reactive –OH^−^ groups (eTi). Then, the samples were introduced into a N_2_-saturated chamber with 7 ml anhydrous pentane, 1.2 ml (3-chloropropyl)triethoxysilane (CPTES) and 0.6 ml diisopropylethylamine (DIEA) (all from Sigma-Aldrich, St. Louis, MO, U.S.A.). Samples were in the CPTES solution for 1 h and periodic 2-minute ultrasonication cycles were applied after every 10 minutes of reaction (eTi-Sil). Coatings of peptides by covalent conjugation with silanes was performed in a 0.1 mM solution of GL13K (GKIIKLKASLKLL-NH_2_) or randomized control peptide GL13KR1 (IGIKLLKSKLKAL-NH_2_) (Peptides International, Louisville, KY, U.S.A) with 0.5 mg/ml Na_2_CO_3_ under argon atmosphere overnight.

### Water Contact Angles (WCA)

Sessile drop contact angle measurements on modified Ti discs were performed using a contact angle analyzer (DM-CE1, Kyowa Interface Science, Niiza-City, Japan) with appropriate software (FAMAS, Kyowa Interface Science, Niiza-City, Japan) after each step of the coating method. Deionized water was used as the wetting liquid with a drop volume of 2 µL.

### Visualization of Fluorescent Labeled Peptides

Fluorescence labeled GL13K-ED-FAM obtained by solid-phase peptide synthesis (purchased from AAPPTec, Louisville, KY, U.S.A.) was covalently coated on the Ti surfaces. Intensity of the surface fluorescence signal was observed by fluorescence microscopy (Eclipse E800, Nikon, Tokyo, Japan).

### X-ray photoelectron spectroscopy (XPS)

XPS was performed (Al Kα x-ray, 1 mm spot size, 35° take-off angle) to obtain survey scans at 1 eV step-size. Peak fittings and quantification of surface chemical composition were conducted using ESCA 2005 software provided with the XPS system (SSX-100, Surface Science Laboratories, U.S.A.).

### Resistance of GL13K Peptide Coatings to Proteolytic Degradation

Fluorescent labeled GL13K was covalently coated (cov-GL13K) or physically adsorbed (pTi-phys-GL13K) on Ti surfaces followed by incubation in saliva at 37°C for 11 days. Pulled unstimulated saliva was freshly collected following stablished protocols [27]. An exemption from IRB approval was granted because saliva was collected from the researcher that performed these tests. Saliva was collected an hour before the initiation of the experiment and an hour before renewing saliva every 2 days. During collection, saliva was kept in a vial surrounded by cracked ice to keep the solution fresh [27]. The saliva was filtered with 0.22 µm filters (Argos Technologies, U.S.A.) before its use. The amount of released peptides in saliva was measured using the Synergy 2 multi-mode microplate reader (Bio-Tek Instruments, Winooski, VT, U.S.A.) by referring to the standard curve. The intensity of the retained peptide on the surface on day 0, 1, 4, 7 and 11 was assessed by fluorescence optical density (OD) with the microplate reader. Then, the OD values of samples at day 1, 4, 7 and 11 were converted to concentration of peptide on the surface by referring to the surface peptide concentration at time  =  0 minutes, which was determined using the Bradford assay (Bio-Rad, Hercules, CA, U.S.A.). The Bradford assay was not used to determine the surface peptide concentration after the surface was immersed in body fluid as unspecific protein adsorption on the peptide-coated surface could potentially interfere with the measurements of this test. The percentage of released peptide was calculated by dividing the amount of peptides released in saliva by the amount of peptides retained on the surface. Homogeneity of the surface fluorescence intensities was also visualized using a fluorescence microscope (Eclipse E800, Nikon, Tokyo, Japan).

### Drip Flow Bioreactor Culture

S. gordonii strain ML-5 were inoculated in 2 ml Bacto Todd-Hewitt broth (BD Biosciences, San Jose, CA, U.S.A.) and cultured overnight at 37°C. Titanium discs were UV sanitized for 10 min then placed into sample holders in the channels (3 discs per channel: eTi, GL13K and GL13KR1) of the drip flow biofilm reactor (BioSurface Technologies Corp., Bozeman, MT, U.S.A) (Figure S1-A in File S1). The first/static phase consisted of overnight bacterial culture under static conditions (Figure S1-B in File S1) where 20 ml of 10^7^ cfu/ml *S. gordonii* were loaded in each channel of the bioreactor. The channel temperature was 37°C. The subsequent second/dynamic phase consisted of 48 h under continuous flowing conditions (Figure S1-C in File S1). During this phase a 10° inclined base was located underneath the bioreactor and a peristaltic pump was used to flow Bacto Todd-Hewitt broth media through the bioreactor channels at a rate of 0.3 ml/min. The flow rate was selected from the low end range of un-stimulated salivary flow rate, 0.1–2 ml/min [28] because at the gingival sulcus where saliva might be in contact with implant surfaces, this is expected to be much lower than the average flow rate in open areas of the mouth. At the end of the experiment, samples were rinsed with 0.9% NaCl solution.

### Regular Culture

Ti discs were UV sanitized in a 48-well plate, and incubated with 1 ml of 10^7^ cfu/ml *S. gordonii* at 37°C on a shaker at 60 rpm for the same total time as the samples in the bioreactor.

### Colony Forming Units (CFU) and ATP Assay

Samples were sonicated in 300 µl 0.9% NaCl for 10 min. 100 µl of the collected solution was mixed with 100 µl of the BacTiter-Glo Microbial Cell Viability kit (Promega, Madison, WI, U.S.A) and the luminescence was measured with a micro-plate luminometer (BioTek, Winooski, VT, U.S.A). Another 100 µl of the collected solution was used for the CFU test. Briefly, 100 µl of the obtained solution was serially diluted 10–10,000 fold. Ten µl of each dilution were plated on Todd-Hewitt Agar plates and incubated overnight at 37°C and the number of CFU was counted.

### Live/Dead Cell Staining

LIVE/DEAD BacLight Bacterial Viability Kit (Invitrogen, Grand Island, NY, U.S.A.) was used to stain bacterial cells. Briefly, equal volumes of live stain (green fluorescence) and dead stain (red fluorescence) were mixed and diluted to 0.3% in water. Ten µl of the diluted fluorescent stains were incubated with the samples collected after bacteria culture for 15 minutes. Samples were transferred to a fluorescence microscope (Eclipse E800, Nikon, Tokyo, Japan) for visualization.

### Scanning Electron Microscopy

Visualization of bacteria and grown biofilms was performed using a field-emission scanning electron microscope (FE-SEM) (6500, JEOL Ltd., Tokyo, Japan). Bacteria were fixed with 2% glutaraldehyde and 0.15% alcian blue in 0.1 M sodium cacodylate buffer (pH 7.4) for 1 h. They were secondarily fixed with 1% OsO4 in 0.1 M sodium cacodylate buffer for 1 h. Then, samples were dehydrated with ethanol solutions at increasing concentrations, critical-point dried, and sputter coated with 5 nm Pt.

### Statistical Analysis

Statistically significant differences (p<0.05) between groups were assessed using one-way ANOVA Tables with LSD post-hoc tests.

## Results and Discussion

### GL13K Peptide coatings

**Figure 1:**
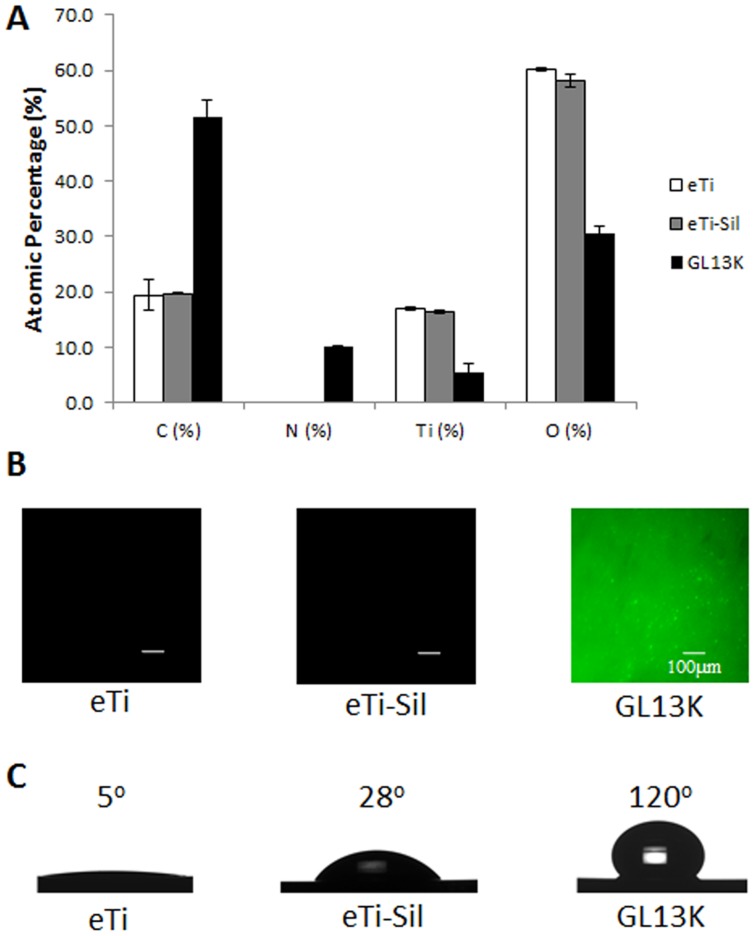
GL13K-peptide coatings characterization. (A) XPS elemental quantification of the main constituents of the GL13K-coated titanium surfaces and controls. The emergence of a strong N peak and increase of the C signal in the GL13K surfaces indicated that the peptides were successfully retained on the titanium surfaces. B) Visualization of fluorescently-labeled GL13K peptides demonstrated the homogeneous distribution of the peptides on the coated surfaces. (C) Water contact angles showed that the coating of the GL13K peptides on the titanium surfaces produced a super-hydrophobic surface.

**Figure 2:**
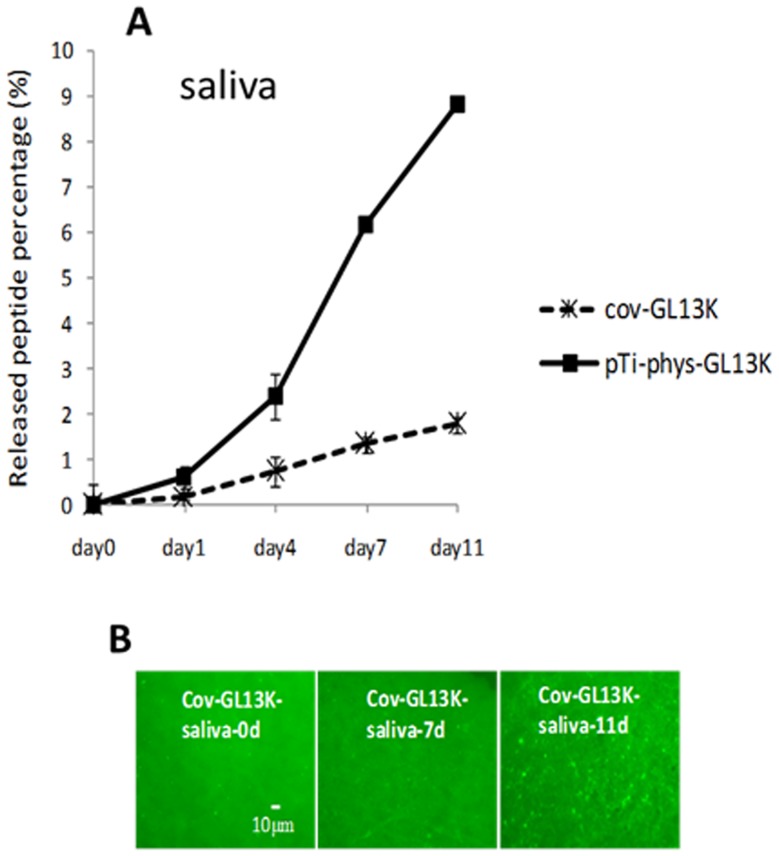
Resistance of GL13K-peptide coating to proteolytic degradation in saliva. A) Percentage of fluorescently-labeled GL13K peptides released after 11days of incubation in saliva from surfaces coated with covalently-bonded (cov-GL13K) or physically adsorbed peptides (pTi-phys-GL13K). The homogeneity of the peptides retained on the coated surfaces was observed by fluorescence microscopy (B). The peptides were not notably degraded or removed from the surface after being exposed to the enzyme-containing biological solution. Error bars are the standard deviation of three samples in each group.

XPS analysis of the GL13K-coated Ti surfaces showed strong nitrogen peaks due to the presence of the aminoacids (Figure 1-A, Table S1 in File S1), suggesting that the peptides were effectively retained on the titanium surface. The GL13K peptides were homogenously distributed on the surface (Figure 1-B) and formed coatings which were highly hydrophobic with water contact angles higher than 120°C (Figure 1-C). Resistance to proteolytic degradation is a crucial property of peptide coatings designed to have sustained activity as they are potentially vulnerable to enzymes and proteases present in saliva. To test the resistance of our coatings to enzyme degradation, we coated fluorescence labeled GL13K peptides onto the surface, followed by immersing the coating in saliva for 11 days. We investigated the percentage of peptide cleaved from the surface to the proteolytic solution. The control group was physically-adsorbed GL13K on plain Ti surfaces. After 11 days of incubation in saliva, the covalently anchored coatings, cov-GL13K showed a 2% peptide release from the surface. Peptides from control surfaces rose to around 9% release to saliva (Figure 2-A). Figure 2-B shows images of the surfaces degraded by saliva for 11 days. Thus, most of the peptides were retained on coatings of cov-GL13K surfaces after a long period of incubation in saliva.

### Antimicrobial Effect of GL13K Coatings

**Figure 3:**
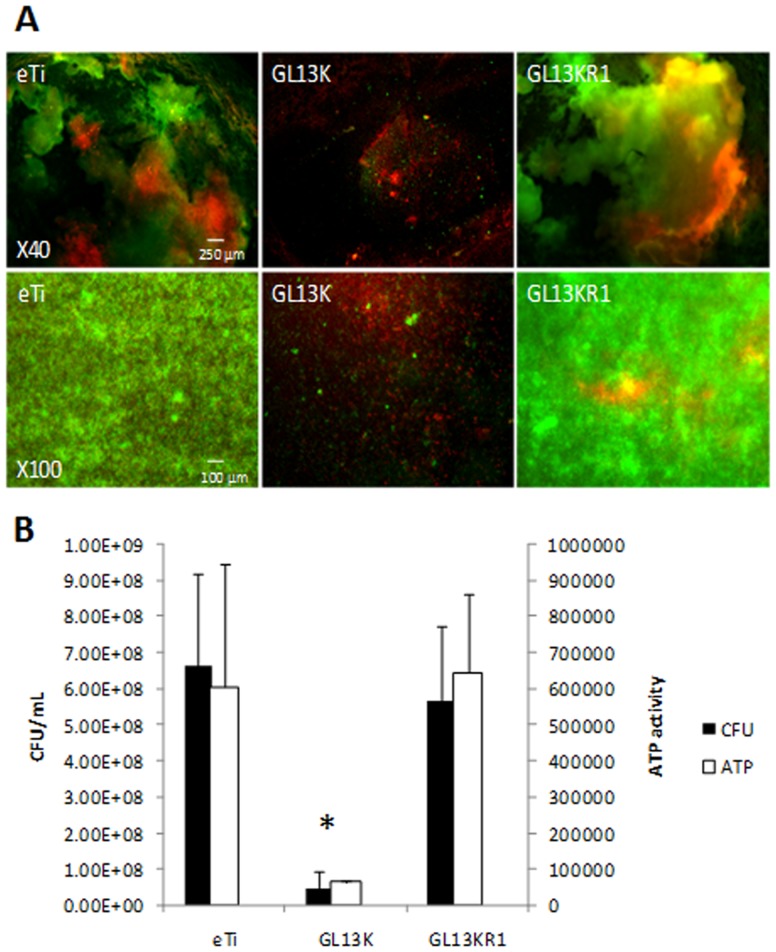
Antimicrobial effects of GL13K-peptide coatings on *S. gordonii* cultured for 3d in the drip flow bioreactor. A) Live/dead cell fluorescence staining; and B) CFU and ATP activity demonstrated the significant (*, p<0.05) and strong antimicrobial effect of the GL13K peptide coatings in comparison to all control surfaces. *S. gordonii bacteria* were killed on the GL13K coated surfaces. Error bars are the standard deviation of at least three samples in each group.

Live/dead cell staining assays revealed that after 3 days of culture in the bioreactor, very thick *S. gordonii* biofilms grew on eTi and eTi coated with the negative control peptide GL13KR1 (Figure 3-A). On the contrary, the GL13K coatings substantially reduced bacterial adhesion and prevented biofilm formation and growth (Figure 3-A). Bacteria adhered to the GL13K-coated surface were predominantly dead, which indicated that the antimicrobial peptide coating exhibited a direct bactericidal effect. Consistent with these results, *S. gordonii* viability and metabolic activity were significantly reduced on GL13K-coated titanium in comparison to the ones on control surfaces; i.e., non-coated eTi and eTi coated with the control peptide GL13KR1 (Figure 3-B).

The bioactivity of the GL13K coating against *S. gordonii* was also observed (data not shown) in overnight cultures in an orbital shaker (60 rpm). Our results here demonstrated that the GL13K-peptide coatings preserved their remarkable antimicrobial effect when tested using the more challenging culture conditions applied with the drip-flow bioreactor system, which more closely simulates in vivo conditions. This suggests that the GL13K-coated surfaces may have clinical application in peri-prosthetic infections.

### Cell Wall Rupture by GL13K-Peptide Coatings

**Figure 4:**
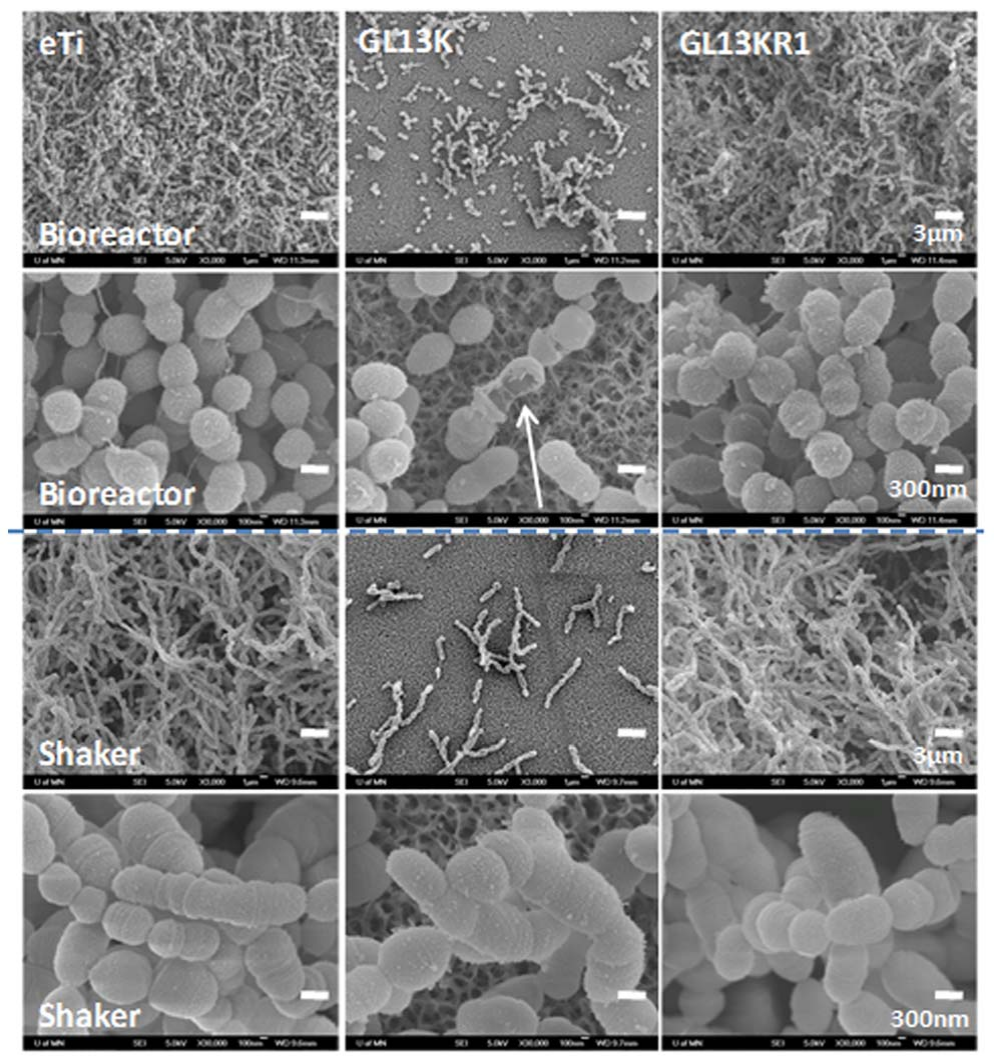
FE-SEM images of *S. gordonii* biofilms grown on the tested surfaces. General view (1^st^ and 3^rd^ rows) and Close-up (2^nd^ and 4^th^ rows) images of surfaces tested in the drip flow bioreactor (1^st^ and 2^nd^ rows) and the orbital shaker (3^rd^ and 4^th^ rows). The biofilms did not form and grow on the GL13K coatings in either of the two culture conditions. However, ruptured bacteria (white arrow) were observed when bacteria were cultured under dynamic conditions in the drip flow bioreactor.

FE-SEM visualization of *S. gordonii* biofilms formed in the bioreactor revealed the morphology, surface topography, integrity, and structure of bacteria and biofilms grown on the different tested surfaces. Low resolution SEM images confirmed the live/dead test results as very thick biofilms were detected on the control surfaces; whereas the GL13K-coated surfaces had very low numbers of bacteria adhered and biofilms did not form (Figure 4, 1^st^ row).

Notably, high resolution FE-SEM pictures of the bacteria that attached to GL13K-peptide coatings revealed that a number of cells had a disrupted cell wall (Figure 4, 2^nd^ row). The cell wall rupture resulted in formation of either holes on the bacterial wall or cracks along circumferential lines of the wall (arrows). Bacteria already showed wall disruption at the initial stages (up to 6 h) after starting the dynamic phase in the drip-flow bioreactor (Figure S2 in File S1). None of the bacteria in biofilms grown on the control surfaces (eTi and GL13KR1) in the bioreactor exhibited this lack of mechanical integrity of their wall.

**Figure 5:**
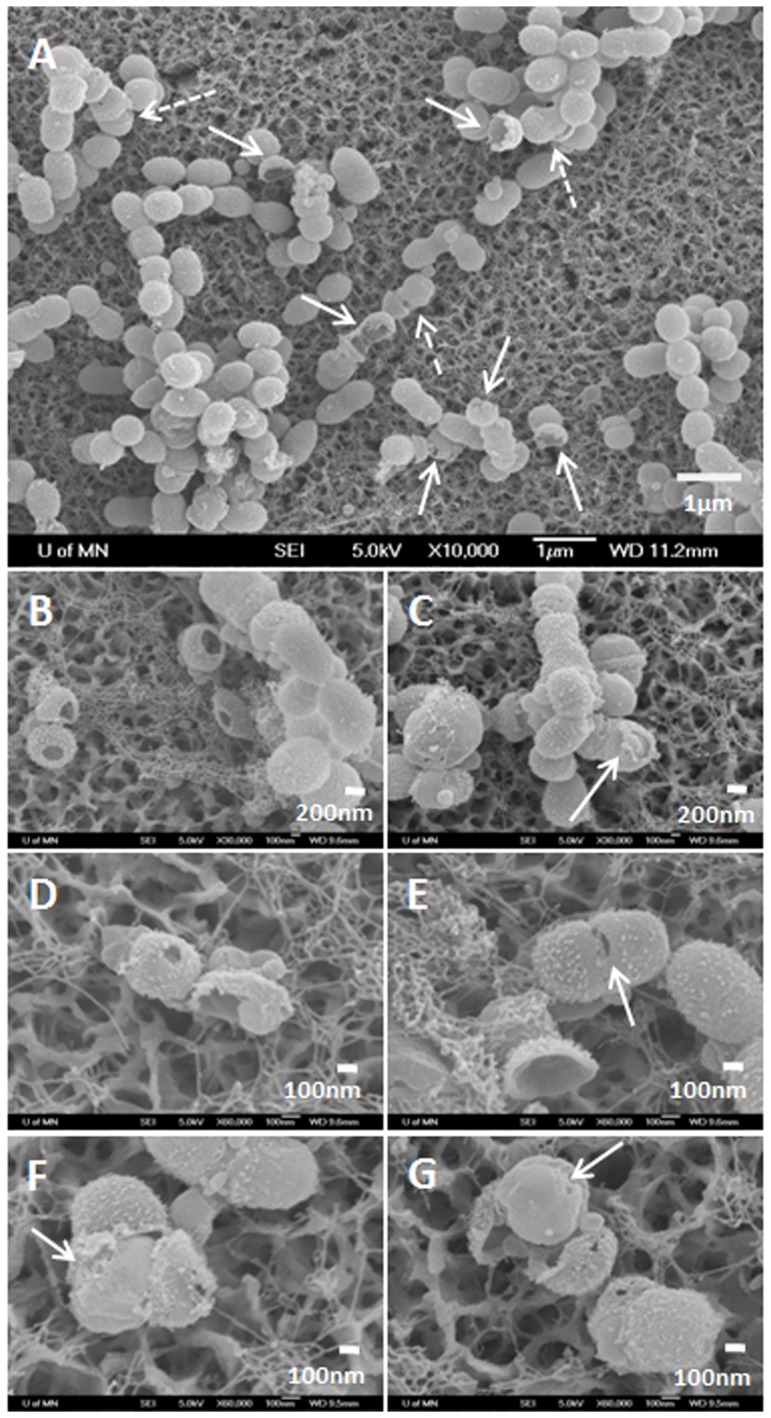
FE-SEM images of the morphology of disrupted *S. gordonii* bacteria wall cultured in the drip flow bioreactor on GL13K-coated surfaces. GL13K peptide coatings ruptured the cell wall of multiple bacteria (A). Two types of broken bacteria were identified: Bacteria with empty shell-like cell wall structures (solid arrows in A). Close up of these emptied broken walls can be observed in B, D, E. Bacteria that showed the protoplast (dashed arrows in A). Close up of bacteria exposing the protoplasts can be observed in C, D, F, and G. Arrows in C and E point to cells with broken walls at the polar and septum areas, respectively. Arrows in F and G point to protoplasts with localized disturbances of the bacterial membrane.

We further investigated the unique occurrence of the observed cell wall rupture by conducting new experiments with the same surface groups and periods of incubation (up to three days), but under mild agitation (60 rpm) conditions. Under these culture conditions, again the GL13K peptide coatings prevented biofilm formation whereas dense biofilms grew on all control surfaces (Figure 4, 3^rd^ row). However, damage to the cell wall of the bacteria was not observed in any of the tested GL13K surfaces (Figure 4, 4^th^ row). All of the evidence demonstrated that the GL13K peptide coating had bactericidal activity and prevented *S. gordonii* biofilm formation and growth presumably due to the peptide's ability to interact with and disrupt the bacterial membrane [29]. However, it was only when in combination with media flow and attendant biological factors of the incubation conditions in the bioreactor system that the GL13K-peptide coating ruptured the cell wall of these Gram positive bacteria. The sustained supply of media in the bioreactor, as compared to the orbital shaker, increased the metabolic activity of the bacteria, resulting in much thicker biofilms with smaller and more actively dividing cells (Figure 4). A more detailed look at the morphological features of the broken bacteria in Figure 5 revealed that the rupture of the cells commonly occurred in their septum and/or polar area (Figure 5-C and Figure 5-E, arrow), which are the areas where enzymatic activity is enhanced to initiate the process of severing the cell during the active phases of bacterial division [30]. This suggests that stimulation of cell division under culture conditions in the bioreactor assisted the activity of GL13K peptides in damaging the bacterial wall, which eventually resulted in the observed wall rupture.

Figure 5 also shows two general types of broken wall morphologies: Rupture of the bacterial cell wall with (dashed arrow) or without (solid arrow) the protoplast (Figure 5-A). Figure 5-B, -D, -E show bacteria with broken cell walls that did not retain their protoplasts. This left empty shell-like structures of the bacterial wall. Figure 5-F, -G show splitting of the cell wall and exposed bacterial protoplast. After enzymatic digestion of the cell wall, the protoplast can be stabilized in an osmotic condition in which cell lysis does not occur. However, it is noteworthy that the cell membrane in bacteria visualized in Figure 5-F, -G show localized morphological disturbances (arrows) that may indicate their compromised function. Approximately 10% of bacteria on GL13K coated surfaces exhibited cell wall rupture. This is an underestimated number because bacteria that ruptured in parts of the cells that are hidden to the FE-SEM view can not be counted and others were washed away during the rinsing and preparation of the samples for SEM visualization. Nevertheless, the bactericidal effects of the GL13K coatings –measured by CFU and visualized in the Live/Dead fluorescence staining images was notably higher than a mere 10%. This suggests that the cell wall rupture is a relevant associated phenomenon but not the only cause for the bacterial death.

The interaction of antimicrobial peptides with bacterial cell membranes has been studied extensively and typically leads to pore formation through one of several proposed mechanisms [31]. It has been recently shown that GL13K interacts with artificial membranes in β-sheet conformations to produce membrane holes [32]. However, the covalently attached GL13K peptides on the titanium surfaces are unlikely to affect the cytoplasmatic membrane of *S. gordonii* in that way as they are unable to move freely and the membrane of Gram positive bacteria is protected by a thick peptidoglycan wall. Thus direct contact of the peptides in the coating with the cytoplasm membrane is unlikely to happen. The rupture of Gram positive bacteria by coated surfaces with antimicrobial peptides has not previously been observed and thus, an alternative model is needed to describe the cell wall rupture. Previous evidence and proposed mechanisms support that cell wall damage and eventual rupture of the Gram positive bacteria can be mediated by a molecular interaction between the GL13K peptides in the coating and wall teichoic acids (WTA) in the bacteria wall. Notwithstanding, the in vivo oral biofilm is a multi-species entity and some bacteria might have extracellular protease activity which could partially hydrolyze peptides. Therefore, the long term stability and the effectiveness of the GL13K coating needs to be further researched.

The peptidoglycan layer of the cell wall that prevents the cell membrane from rupturing under osmotic pressures [33] is also actively modified by glycosidases and peptidases, the so-called autolysins to allow cell division. Autolysins need to be under strict control since lysis and cell death would follow any unrestricted activity. It has been suggested that WTA play an essential role in restricting the activity of autolysins [34]–[36]. WTA are less concentrated around the septum, where the next cell division will occur and play a key role in regulating the cationic environment of the cell [37]. WTA have strong affinity for cationic antimicrobial peptides because of their highly-anionic property [34], [38]. The positive surface created by the disks could displace the autolysins from the WTA and make them free to attack the less protected areas around the cell at the septum, resulting in the observed rupturing of the cell. Biswas et al. recently suggested that the negatively charged WTA phosphate groups trap protons in the cell wall, creating an acidic environment that keeps autolysin activity at a low level [39]. The cationic antimicrobial peptides may strongly interact with the phosphate groups of the WTA leading to the release of protons. The resulting increased local pH activates autolysin, which would weaken the cell wall by breaking down glycosidic bonds and peptide crosslinks. A similar argument has been made recently for triggering cell lysis in *Staphlococcus aureus* by the antimicrobial peptide rBPI21 [40], although the effect was not associated with peptides anchored on a surface.

In this work we have shown for the first time that antimicrobial GL13K peptide coatings induced cell wall rupture under dynamic salivary-flow rate conditions on a drip flow bioreactor, acting differently from GL13K in solution or tested under regular culture conditions where exposure did not result in cell lysis [20], [21]. This unique observation may lead to the discovery of new mechanisms by which antimicrobial peptide coatings kill Gram positive bacteria.

## Conclusions

The covalently-immobilized GL13K antimicrobial peptide coating had excellent antimicrobial activity when exposed to *S. gordonii* cultures in a drip flow biofilm reactor system. These culturing conditions in combination with the activity of the GL13K peptide coatings resulted in rupture of the cell wall of Gram positive bacteria.

## Supporting Information

File S1 **Supporting Figures and Table. Table S1.** **XPS elemental quantification of modified Ti surfaces. Figure S1. Drip flow biofilm reactor system.** A) Ti samples with or without coatings were loaded in each of the four channels of the bioreactor. B) S. gordonii bacteria were cultured overnight under static conditions followed by C) 48 h culture with a continuous media flow rate. **Figure S2. FE-SEM images of bacteria cultured for different periods in the drip flow bioreactor on GL13K-coated surfaces.** Bacteria showed cell wall rupture on the GL13K surface after 6, 24, 30 and 48 h of continuous dynamic culture conditions. Rupture of the cell wall occurred at early stages after initiating the flow in the bioreactor (6 h).
(DOC)
