# Supplementary material for: Antimicrobial GL13K Peptide Coatings Killed and Ruptured the Wall of Streptococcus gordonii and Prevented Formation and Growth of Biofilms
Source: PLoS One. 2014 Nov 5;9(11):e111579. doi: 10.1371/journal.pone.0111579 (PMC4221044; doi:10.1371/journal.pone.0111579)
Supplement: File S1 — Supporting Figures and Table. Table S1. XPS elemental quantification of modified Ti surfaces. Figure S1. Drip flow biofilm reactor system. A) Ti samples with or without coatings were loaded in each of the four channels of the bioreactor. B) S. gordonii bacteria were cultured overnight under static conditions followed by C) 48 h culture with a continuous media flow rate. Figure S2. FE-SEM images of bacteria cultured for different periods in the drip flow bioreactor on GL13K-coated surfaces. Bacteria showed cell wall rupture on the GL13K surface after 6, 24, 30 and 48 h of continuous dynamic culture conditions. Rupture of the cell wall occurred at early stages after initiating the flow in the bioreactor (6 h). (DOC) [file pone.0111579.s001.doc]

**Antimicrobial GL13K peptide coatings cause rupture of Gram positive bacteria**

Xi Chen, Helmut Hirt, Yuping Li, Sven-Ulrik Gorr, Conrado Aparicio

***Supplementary Data***

**Table S1. XPS elemental quantification of modified Ti surfaces.**

| **Surface** | **Elemental composition** | | | | | | |
| --- | --- | --- | --- | --- | --- | --- | --- |
|  | Si (%) | Cl (%) | C (%) | N (%) | Ti (%) | O (%) | Bal. (%) |
| eTi | 0±0 | 0±0 | 19.4±2.9 | 0±0 | 16.8±0.7 | 60.1±2.6 | 3.7±1.2 |
| eTi-Sil | 1.1±0.5 | 1.3±0.4 | 19.6±0.1 | 0±0 | 16.3±0.3 | 58.1±1.1 | 3.5±0.7 |
| GL13KR1 | 0±0 | 0.1±0.1 | 40.9±2.8 | 9.4±0.3 | 10.1±0.8 | 37.8±1.0 | 1.6±0.3 |
| GL13K | 0.4±0.4 | 0.8±0.2 | 51.5±3.2 | 10.1±0.3 | 5.5±1.4 | 30.5±1.3 | 1.2±0.5 |


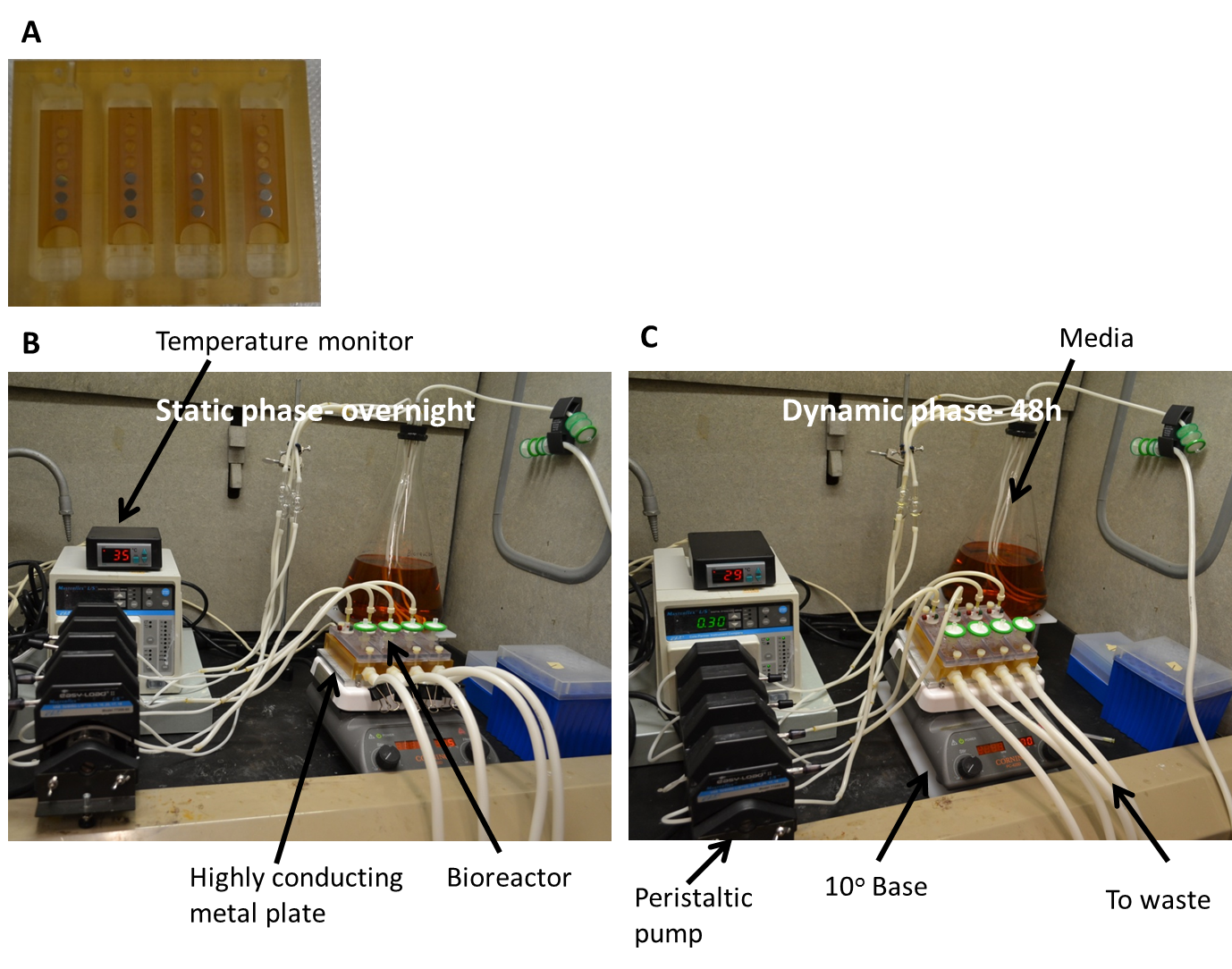


**Figure S1**. **Drip flow biofilm reactor system.** A) Ti samples with or without coatings were loaded in each of the four channels of the bioreactor. B) *S. gordonii bacteria* were cultured overnight under static conditions followed by C) 48h cultured with a continuous media flow rate.


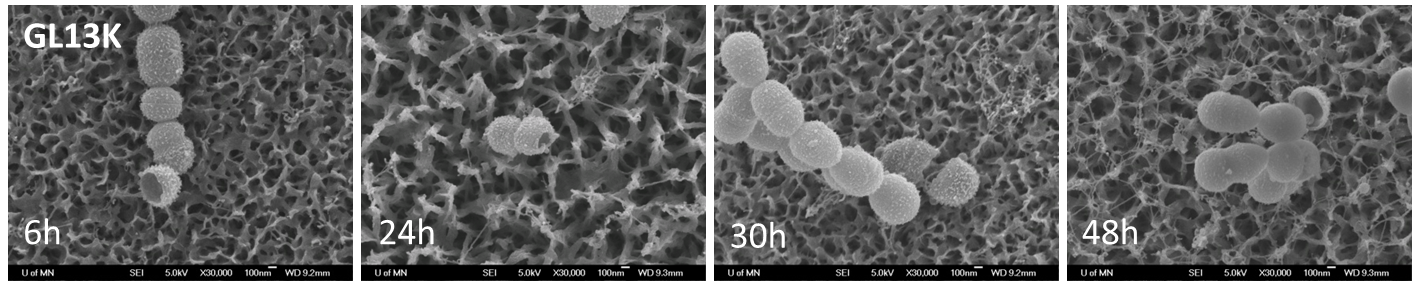


Figure S2. SEM images of bacteria cultured for different periods in the drip flow bioreactor on GL13K-coated surfaces. Bacteria showed cell wall rupture on the GL13K surface after 6, 24, 30 and 48 h of continuous dynamic culture conditions. Rupture of the cell wall occurred at early stages after initiating the flow in the bioreactor (6h).
